# Case Report: Zanubrutinib for refractory immune thrombotic thrombocytopenic purpura

**DOI:** 10.3389/fimmu.2026.1895825

**Published:** 2026-10-14

**Authors:** Adrián Dante De Angelis, Annatina Schnegg-Kaufmann, Elisavet Moutzouri, Katarzyna Aleksandra Jalowiec, Milos Ratknic, Johanna A. Kremer Hovinga, Ioannis Chanias

**Affiliations:** 1 Department of Hematology and Central Hematological Laboratory, Inselspital, Bern University Hospital, University of Bern, Bern, Switzerland; 2 Centre for Oncology and Hematology, Thun-Spiez-Berner Oberland, Spital STS AG, Thun, Switzerland; 3 Department for BioMedical Research, University of Bern, Bern, Switzerland; 4 Department of Hematology, HFR Hôpital Fribourgeois, Fribourg, Switzerland; 5 Centre of Oncology/Haematology and Transfusion Medicine, Cantonal Hospital Aarau, Aarau, Switzerland; 6 Department of Hematology, Bone Marrow Transplantation Unit and Institute of Cell Therapy, University Hospital of Patras, Rio, Greece

**Keywords:** autoimmunity, BTK inhibition, chronic, immune TTP, relapse/refractory

## Abstract

Immune-mediated thrombotic thrombocytopenic purpura (iTTP) results from severe ADAMTS13 deficiency and may relapse despite modern therapy, with survival exceeding 95% in contemporary cohorts. We report the case of a 43-year-old patient with multiple clinical and ADAMTS13 relapses over 15 years, who developed intolerance or had incomplete responses to several immunosuppressive agents, including anti-CD20 and anti-CD38 therapies. Splenectomy was considered high risk due to significant cardiac comorbidity. Off-label zanubrutinib, a second-generation Bruton tyrosine kinase (BTK) inhibitor licensed locally for chronic lymphocytic leukemia (CLL) was initiated after the sixth relapse, which was a clinical one, simultaneously with plasma exchange (PEX) and corticosteroids. Following initiation, ADAMTS13 activity rose, platelet counts normalized, and the patient has remained in clinical remission for 20 months under zanubrutinib. This case illustrates the feasibility of BTK inhibition in selected, treatment-limited iTTP.

## Introduction

Thrombotic thrombocytopenic purpura (TTP) is characterized by microangiopathic hemolysis, thrombocytopenia, and ischemic organ injury resulting from severe ADAMTS13 activity deficiency of <10%, most often immune-mediated (1). Standard-of-care therapy—plasma exchange (PEX), immunosuppression, and caplacizumab—achieves survival >95% in recent studies (2). Relapses may be clinical (thrombocytopenia ±hemolysis) or laboratory/ADAMTS13 only (activity <20% without thrombocytopenia) (3). The Bruton tyrosine kinase (BTK) pathway is central in B-cell signaling and autoantibody production, and BTK inhibitors (BTKis) are under investigation in autoimmune diseases, including immune thrombocytopenia (ITP) (4). Clinical use in immune-mediated TTP (iTTP) has not been reported. Zanubrutinib, a second-generation BTKi with improved safety over ibrutinib, is approved for B-cell malignancies (5). We describe a 43-year-old man (white race) with multiple clinical and ADAMTS13 relapses of iTTP, who was intolerant or refractory, or showed only partial responses to conventional therapies. In order to avoid splenectomy, we attempted an off-label therapy with the second-generation BTKi zanubrutinib, achieving sustained remission.

## Case report

The patient first presented in 2009 with nausea, vomiting, and anorexia. Laboratory evaluation revealed microangiopathic hemolytic anemia accompanied by renal, hepatic, and cardiac impairment. ADAMTS13 activity was <5% with a detectable inhibitor. His prior medical history was notable for obesity and arterial hypertension, and at the time of presentation, he was additionally diagnosed with hypertrophic cardiomyopathy. Family history was unremarkable, with no known family history of iTTP. Psychosocial history was also unremarkable. He achieved a partial remission after PEX, corticosteroids, and rituximab. In 2015, the first clinical relapse was treated similarly, but rituximab had to be discontinued after serum-sickness-like reaction with rash, fever, arthralgias, and systemic inflammation; despite this, remission was achieved.

In November 2020, a second (ADAMTS13) relapse occurred (**Figure 1**). Caplacizumab—though guideline-recommended—was unavailable in Switzerland at that time due to reimbursement restrictions. Obinutuzumab was attempted in December but stopped immediately due to a serum-sickness-like reaction. From February to April 2021, daratumumab was administered weekly for 8 weeks, leading to complete remission. In January 2022, a third relapse in the setting of ADAMTS13 relapse occurred, and a retreatment with daratumumab followed by 6 months of monthly maintenance achieved only partial remission with ADAMTS13, 20%–30%. Therapy was stopped at the patient’s request due to intolerable fatigue, and after 3 months in October 2022, a clinical relapse (fourth) with thrombocytopenia and measurable ADAMTS13 inhibitor (>2 BU/mL) occurred. PEX, corticosteroids, and cyclophosphamide induced partial remission, but in May 2023, ADAMTS13 fell below 10% with new thrombocytopenia (fifth relapse). A second obinutuzumab attempt—after negative skin testing—produced an initial ADAMTS13 rise to 83%, but a severe serum-sickness syndrome recurred before the second infusion, requiring hospitalization and drug discontinuation. A B-cell subset flow cytometry was performed, showing a reduced peripheral B-cell number (40/µL; 2.0% of lymphocytes), with a marked relative expansion of plasmablasts (17.4% of B cells; 6.96/µL) and transitional B cells (29.6%; 11.84/µL). Corticosteroids alone given from June until August 2023 induced a temporary remission, which lasted only 3 months until October, when the sixth relapse (ADAMTS13 relapse) with measurable ADAMTS13 inhibitor (1.6 BU/mL) occurred. This was treated with bortezomib in December 2023, without response, and the patient suffered an ST-elevation myocardial infarction shortly thereafter. Subsequently, mycophenolate mofetil was initiated, but the situation worsened with the occurrence of thrombocytopenia, an increase in LDH, and undetectable levels of ADAMTS13 with high titers of ADAMTS13 inhibitor (>2 BU/mL) in July 2024. The patient’s only symptom was malaise, and the physical examination was unremarkable. At this moment, and with no effective or tolerable immunosuppressive options, splenectomy was considered. To avoid surgery and considering the cardiovascular risk factors, we initiated an off-label trial of the BTKi zanubrutinib in August, with the intention of preventing the progression of ADAMTS13 relapse to clinical relapse, chosen for its favorable cardiac safety compared with ibrutinib (6). After a few days of the beginning of a new treatment with PEX and high-dose corticosteroids, zanubrutinib 320 mg once daily was added on 10 August 2024, together with PEX and corticosteroids. This dose corresponds to the approved dose for chronic lymphocytic leukemia (CLL) and was administered off-label in this setting, in the absence of established dosing recommendations for zanubrutinib in iTTP. A prompt response was observed, with ADAMTS13 activity becoming detectable within 5 days and platelet counts normalizing within 2 weeks. From September 2024 onward, ADAMTS13 activity remained consistently above 50%. This sustained hematologic and biochemical response allowed discontinuation after nine sessions of PEX and subsequent tapering and withdrawal of corticosteroids after 7 weeks. During treatment, in October 2025, approximately 1 year after zanubrutinib initiation, the patient developed worsening of pre-existing arterial hypertension, requiring adjustment of antihypertensive therapy. Type 2 diabetes mellitus had already been diagnosed prior to zanubrutinib initiation in August 2024; however, glycemic control subsequently worsened, requiring initiation of oral antihyperglycemic therapy. Given the sustained response and the emerged adverse events, zanubrutinib was gradually tapered after more than 1 year of treatment. The dose was reduced from 320 to 240 mg/day on 3 October 2025 to 160 mg/day on 11 December 2025 and to 80 mg/day on 17 January 2026 before being discontinued on 6 March 2026. A clinical relapse occurred on 16 April 2026, approximately 6 weeks after zanubrutinib discontinuation. No ADAMTS13 inhibitor was detectable at relapse. Zanubrutinib was restarted at 320 mg/day on the same day, accompanied by three PEX sessions on 17 April, 20 April, and 22 April 2026. This rechallenge resulted in a second complete remission.

**Figure 1:**
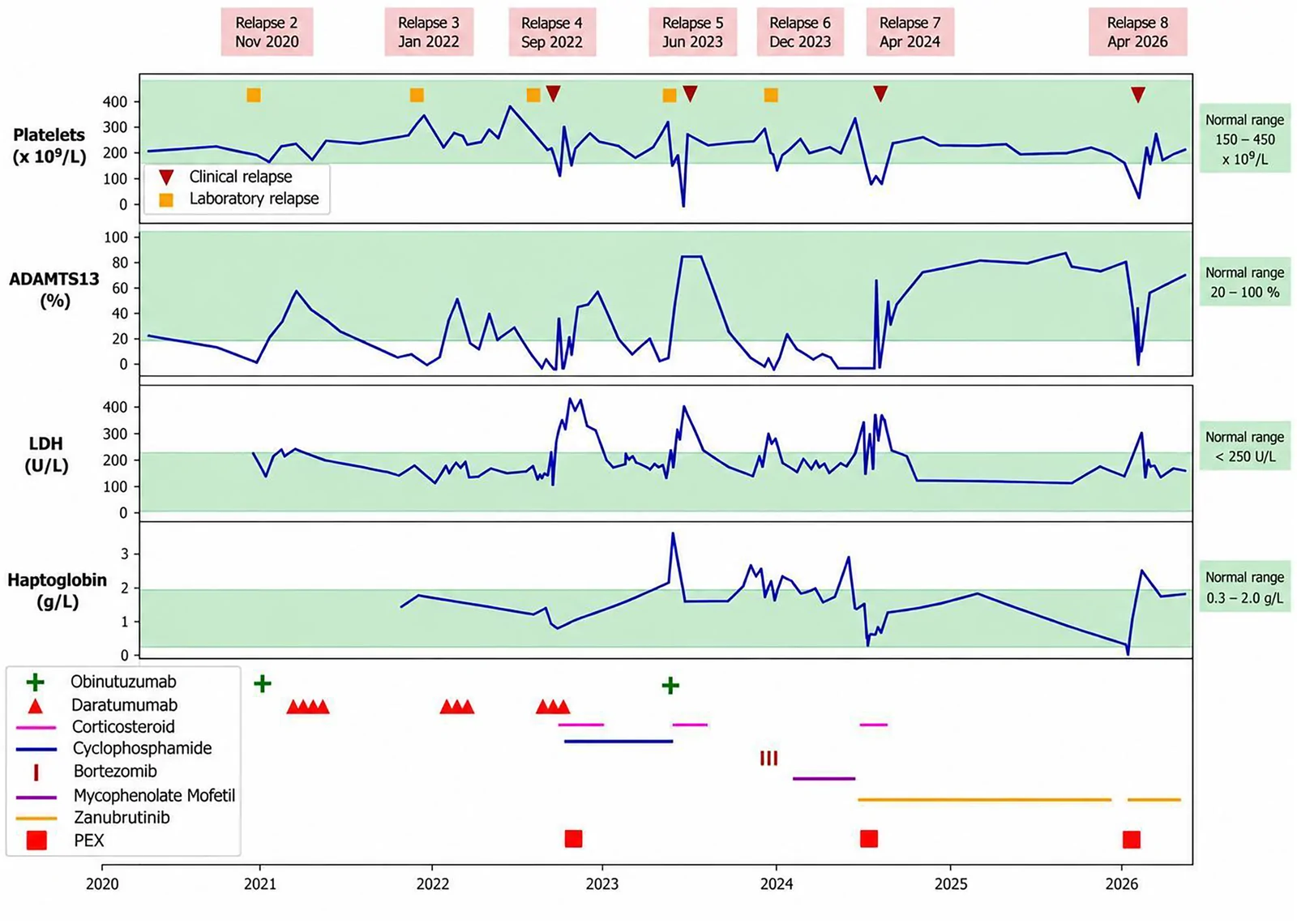
Longitudinal course of platelet count, ADAMTS13 activity, LDH, haptoglobin, and treatment from the second relapse onward. Platelet count, ADAMTS13 activity, lactate dehydrogenase (LDH), and haptoglobin levels are shown over time from 2020 to 2026. Green shaded areas indicate the respective reference ranges. Clinical relapses are indicated by red triangles and laboratory relapses are denoted by orange squares. The lower panel depicts the timing and duration of the administered treatments. At the second relapse in November 2020, the patient received obinutuzumab followed by eight weekly doses of daratumumab. At the third relapse in January 2022, daratumumab was administered weekly for four doses, followed by monthly maintenance therapy. At the fourth relapse in September 2022, four weekly doses of daratumumab were administered without response, followed in October 2022 by therapeutic plasma exchange (PEX), corticosteroids, and cyclophosphamide. At the fifth relapse in June 2023, the patient received a single dose of obinutuzumab combined with prednisone. The sixth relapse in December 2023 was treated with bortezomib and dexamethasone. During the seventh relapse in 2024, mycophenolate mofetil was initiated in April, followed by dexamethasone 40 mg daily for 4 days in July, both without response. PEX, prednisone 100 mg, and zanubrutinib were subsequently initiated in July/August 2024, resulting in a rapid response. At the eighth relapse in April 2026, PEX was performed and zanubrutinib was restarted. PEX, therapeutic plasma exchange.

## Discussion

This case illustrates the management challenges in treatment-limited iTTP, a subgroup distinct from standard relapsing disease. Relapses are frequent in iTTP, with up to 30% of patients experiencing relapse in 2 years and up to 40% in 5 years (1). Besides decreased ADAMTS13 levels as the main risk factor, a cohort study identified age of <25 years, non-O blood group, previous relapses, and not receiving rituximab at first presentation as potential risk factors for relapse (7). For the management of ADAMTS13 relapse, preemptive administration of rituximab is advised, though there is no consensus regarding dosing and administration frequency and our patient was unable to tolerate additional infusions of an anti-CD20 therapy. In our patient, further anti-CD20 therapy was limited by recurrent serum-sickness-like reactions to rituximab and obinutuzumab. Daratumumab initially induced a complete remission, but subsequent retreatment resulted only in a partial response, with ADAMTS13 activity remaining at 20%–30%, and was ultimately discontinued because of intolerable fatigue. Thus, the therapeutic limitations in this patient reflected a combination of treatment intolerance and incomplete or transient responses to conventional immunosuppressive approaches. Clinical relapse should be treated with therapeutic PEX, immunosuppression, and—if available—administration of caplacizumab. Alternatively successful treatment with caplacizumab only has been reported in patients who lack severe symptoms and severe thrombocytopenia (8). However, this option was not available at our institution at the time of the last clinical relapse as this medication could not be administered in Switzerland as it was not reimbursed. Our patient experienced several clinical and ADAMTS13 relapses, and there are limited data and consensus on the management of patients who received multiple treatments. Given the insufficient response to PEX and high-dose corticosteroids, we evaluated the use of BTKi, analogueanalogous to the success of other BTKis in autoimmune hematological diseases, such as rilzabrutinib in the therapy of previously treated patients with relapsing ITP, as an ultimate measure to avoid splenectomy (9).Despite guideline recommendations supporting splenectomy as a second- or third-line option for relapsing or refractory iTTP, in this case, we chose to defer splenectomy based on patient-specific risks and emerging therapeutic alternatives. Relapse rates after splenectomy range from 15% to 50% in published series, depending on patient selection, duration of follow-up, and definitions of relapse (10).

The rapid rise in ADAMTS13 within 4 days is difficult to attribute solely to BTK inhibition because zanubrutinib was initiated concurrently with PEX and high-dose corticosteroids with a total of nine PEX sessions performed before PEX was discontinued. BTK-dependent plasmablast suppression would not be expected to occur this quickly, as seen in iTP and wAIHA trials, where responses typically require weeks. Possible explanations include the following: (1) delayed cumulative effect of PEX + corticosteroids, as ADAMTS13 clearance kinetics vary widely; (2) reduction of anti-ADAMTS13 antibodies through continued PEX with a threshold effect shortly after zanubrutinib initiation; and (3) contribution of BTK inhibition, though mechanistically less likely to exert a rapid effect. We therefore interpret the timing as an association rather than proof of causality. Nonetheless, the sustained remission for more than 12 months with persistent ADAMTS13 recovery, followed by clinical relapse approximately 6 weeks after zanubrutinib discontinuation and renewed complete remission after zanubrutinib rechallenge together with three PEX sessions, supports the plausibility that BTK inhibition contributed meaningfully to disease control.

B-cell phenotype is altered at acute iTTP with decreased transitional cells and post-germinal center (post-GC) memory B cells and increased plasmablasts compared with healthy controls (11). A marked plasmablast expansion was observed in our patient in June 2023 (17.4% of B cells; 6.96/µL). However, the overall B-cell phenotype did not fully reproduce the previously reported pattern, as transitional B cells were increased rather than decreased. These findings should be interpreted cautiously given the single-patient setting, the timing of sampling in relation to disease activity, and the effects of preceding immunomodulatory therapies.

BTK is a non-receptor tyrosine kinase essential to the B-cell receptor (BCR) signaling pathway, which orchestrates B-cell development, activation, and survival. Beyond its foundational role in B-cell biology, BTK is also involved in innate immune signaling, linking it to the pathogenesis of various autoimmune disorders. This dual functionality has positioned BTK as a compelling therapeutic target in both hematologic malignancies and immune-mediated diseases because BTKis block the differentiation of naïve B to cells, plasmablasts, and plasma cells. BTK inhibition has emerged as a therapeutic strategy across several autoimmune diseases, reflecting the role of BCR signaling and Fc receptor-mediated activation in autoantibody production and immune effector mechanisms. However, among these indications, ITP provides the most direct clinical precedent for BTK inhibition in an antibody-mediated hematologic disorder (12, 13). Rilzabrutinib, a reversible BTKi, has demonstrated clinical activity in ITP, supporting the potential relevance of BTK inhibition in antibody-mediated platelet disorders and providing a biologically and clinically relevant precedent for its investigation in iTTP (14).

Among approved BTKis, zanubrutinib, a second-generation agent, is indicated for several B-cell malignancies including mantle cell lymphoma, Waldenström macroglobulinemia, and CLL (5). It offers improved selectivity and a more favorable safety profile compared to first-generation inhibitors (6). These safety advantages support its use in patient populations where tolerability is paramount, potentially including investigational or off-label use in autoimmune diseases (4). However, unlike the BTKis mentioned above, it has no established evidence base in autoimmune diseases.

As iTTP is a rare disease with an incidence of approximately 3 per 1 million adults per year, the prevalence of patients with refractory and recurrent course is even smaller (15). These patients are at high risk of organ complications and related mortality requiring expensive, often parenterally administered immunosuppressive therapies, all of which greatly impacts their quality of life and raises health costs. This report is inherently limited by its single-patient nature and cannot establish a causal relationship between BTK inhibition and the observed response. Furthermore, zanubrutinib trough levels and BTK occupancy were not assessed, limiting pharmacokinetic and pharmacodynamic interpretation of the observed response, dose tapering, and subsequent relapse after treatment discontinuation.

Nevertheless, our patient reported that living with recurrent iTTP and undergoing multiple treatments over many years had a significant impact on his quality of life. He expressed that achieving a sustained remission with zanubrutinib was important to him, particularly as it allowed him to avoid splenectomy. He reported good tolerability of the medication and was satisfied with the clinical response.

In conclusion, the sustained response and subsequent response to rechallenge support further systematic investigation of BTK inhibition in relapsing and treatment-refractory iTTP, ideally in prospective, international clinical trials.

## Data Availability

The original contributions presented in the study are included in the article/supplementary material. Further inquiries can be directed to the corresponding author.
